# Maximizing Oral Health Outcomes of Aboriginal and Torres Strait Islander People With End-stage Kidney Disease Through Culturally Secure Partnerships: Protocol for a Mixed Methods Study

**DOI:** 10.2196/39685

**Published:** 2022-12-16

**Authors:** Sneha Sethi, Brianna Faye Poirier, Joanne Hedges, Zell Dodd, Priscilla Larkins, Cindy Zbierski, Stephen P McDonald, Shilpanjali Jesudason, Lisa Jamieson, AKCTION Group

**Affiliations:** 1 Australian Research Centre for Population Oral Health Adelaide Dental School University of Adelaide Adelaide Australia; 2 Yadu Health Aboriginal Corporation Ceduna Australia; 3 Umoona Tjuagku Health Service Aboriginal Corporation Coober Pedy Australia; 4 Nunyara Aboriginal Health Service Whyalla Australia; 5 Australia and New Zealand Dialysis and Transplant Registry Faculty of Health and Medical Sciences University of Adelaide Adelaide Australia; 6 Central Northern Adelaide Renal and Transplantation Service Faculty of Health and Medical Sciences University of Adelaide Adelaide Australia; 7 See Acknowledgments

**Keywords:** end-stage kidney disease, Aboriginal and Torres Strait Islander health, oral health, health promotion, cultural security, health services, Indigenous health

## Abstract

**Background:**

Dialysis for end-stage kidney disease (ESKD) is the leading cause of hospitalization among Aboriginal and Torres Strait Islander individuals in Australia. Poor oral health is commonly the only obstacle preventing Aboriginal and Torres Strait Islander people with ESKD in Australia from receiving kidney transplant.

**Objective:**

This study aims to improve access, provision, and delivery of culturally secure dental care for Aboriginal and Torres Strait Islander individuals with ESKD in South Australia through the following objectives: investigate the facilitators of and barriers to providing oral health care to Aboriginal and Torres Strait Islander patients with ESKD in South Australia; investigate the facilitators of and barriers to maintaining oral health among Aboriginal and Torres Strait Islander people with ESKD in South Australia; facilitate access to and completion of culturally secure dental care for Aboriginal and Torres Strait Islander individuals with ESKD and their families; provide oral health promotion training for Aboriginal health workers (AHWs) at each of the participating Aboriginal Community Controlled Health Services, with a specific emphasis on oral health needs of patients with ESKD; generate co-designed strategies to better facilitate access to and provision of culturally secure dental services for Aboriginal and Torres Strait Islander people living with ESKD; and evaluate participant progress and AHW oral health training program.

**Methods:**

This collaborative study is divided into 3 phases: exploratory phase (baseline), intervention phase (baseline), and evaluation phase (after 6 months). The exploratory phase will involve collaboration with stakeholders in different sectors to identify barriers to providing oral health care; the intervention phase will involve patient yarns, patient oral health journey mapping, clinical examinations, culturally secure dental care provision, and strategy implementation workshops; and the evaluation phase will involve 6-month follow-up clinical examinations, participant evaluations of dental care provision, and AHW evaluation of oral health training.

**Results:**

Stakeholder interviews were initiated in November 2021, and participant recruitment commenced in February 2022. The first results are expected to be submitted for publication in December 2022.

**Conclusions:**

Expected outcomes will identify the burden of oral disease experienced by Aboriginal and Torres Strait Islander people with ESKD in South Australia. Qualitative outcomes are expected to develop a deeper appreciation of the unique challenges regarding oral health for individuals with ESKD. Through stakeholder engagement, responsive strategies and policies will be co-designed to address participant-identified and stakeholder-identified challenges to ensure accessibility to culturally secure dental services for Aboriginal and Torres Strait Islander individuals with ESKD.

**International Registered Report Identifier (IRRID):**

PRR1-10.2196/39685

## Introduction

### Background

Oral health is a fundamental indicator of overall health and well-being [1]. Despite the importance of oral health, oral disease is commonly experienced by children and adults around the world, with periodontal disease and dental caries being the 2 leading indicators of poor oral health [1]. Periodontal disease is an inflammatory and infectious condition of the supporting bone and soft tissues around teeth, characterized by gingival bleeding, receding gum tissues, and tooth mobility [2]. Dental caries is a result of prolonged carbohydrate metabolism catalyzed by acidogenic bacteria, leading to demineralization of tooth structures [3]. When left untreated, dental caries and periodontal disease can cause substantial pain and fatal infections that spread to other areas of the head and neck [1]. According to the Global Burden of Disease 2017 [4], untreated dental caries in permanent teeth is the most common health condition, and >530 million children worldwide experience dental caries of primary teeth. Severe periodontal disease affects approximately 10% of the global population [4,5].

The direct effect of poor oral health includes pain, functional impairment, and esthetic concerns. However, the indirect effects can be more detrimental to overall health and include difficulties in eating, chronic inflammatory conditions, poor quality of life, and systemic infections that aggravate other comorbidities [6]. In Australia, Aboriginal and Torres Strait Islander communities experience a disproportionate burden of oral disease in comparison with their non-Indigenous counterparts, across all age groups and oral health indicators [7]. This inequity has been attributed to several factors, including the impacts of colonization and assimilation policies [8], neoliberal policies and ideologies [9], experiences of racism in health settings [10], inaccessibility of health services [11], high costs of dental care [12], and inability of mainstream services to meet the health needs of Aboriginal and Torres Strait Islander people [13,14]. Notably, Aboriginal and Torres Strait Islander communities receive less preventive dental care than non-Indigenous Australians [15].

The relationship between oral health and end-stage kidney disease (ESKD) and its precursor, chronic kidney disease (CKD), is well understood. The biological pathway between the mouth and kidney is primarily via the inflammatory response to oral pathogens entering the circulation through bleeding gums and stimulating C-reactive protein production by the liver. Poor oral health commonly experienced by patients with CKD has been attributed to endocrinological, uremic, metabolic, and immunological imbalances [16], and it is evidenced by changes in patients’ teeth [17-21], oral mucosa [22-26], bone [18,27], periodontium [28-30], salivary glands [23,31,32], and tongue [33]. A study in Australia’s Northern Territory reported that Aboriginal and Torres Strait Islander individuals with kidney disease exhibited more indicators of poor oral health when compared with both non-Indigenous populations and general Aboriginal and Torres Strait Islander populations [34].

Oral health has a profound effect on the well-being of patients with kidney disease not only because of the biological pathways but also because optimal oral health is a prerequisite for kidney transplant [35]. For patients with ESKD, poor oral health can increase delays in kidney transplant wait-listing or completely prevent individuals from receiving a kidney transplant. Oral health is necessary for successful kidney transplant because of the infective and inflammatory environments created by dental disease in the body, which have the potential to lead to fatal septic conditions among patients who are immunocompromised, such as those with kidney disease. In addition to the commonly experienced challenges in maintaining oral health among Aboriginal and Torres Strait Islander people, individuals with ESKD face unique circumstances that make optimal oral health more difficult to achieve. For example, dialysis is time consuming; the average patient has 4- to 6-hour sessions, 3 to 4 times per week, meaning that scheduling dental appointments can be difficult. Studies from Central Australia estimated the prevalence of severe periodontal disease among Aboriginal and Torres Strait Islander people with ESKD to be 54%, which is approximately 20 times the national prevalence reported in the 2017 to 2018 National Survey of Adult Oral Health.

According to the Australian Bureau of Statistics, in 2019, 3.4% of Australia’s population identified as Aboriginal and Torres Strait Islander but represents >7% of patients receiving treatment for kidney disease nationally [36]. In 2019, 157 out of 2091 (7.5%) South Australians receiving dialysis identified as Aboriginal and Torres Strait Islander, despite comprising only 2% of the total South Australian population. Of the 1100 Australians who received a kidney transplant in 2019, 33 (3%) were Aboriginal and Torres Strait Islander patients [36]. It has also been estimated that 50% of the non-Indigenous population with ESKD has received a renal transplant, whereas only 13% of the Aboriginal and Torres Strait Islander people with kidney disease have received a transplant [37]. Dialysis in South Australia’s public sector is provided through the Royal Adelaide Hospital’s Central and Northern Adelaide Renal and Transplantation Service (CNARTS) and Flinders Medical Centre. Aboriginal and Torres Strait Islander people in South Australia generally receive primary health care, including kidney-related health care, through Aboriginal Community Controlled Health Services (ACCHS). The ACCHS facilitates transport and accommodation [37] for off-site services; counseling; and management of other chronic care needs, such as type 2 diabetes. Owing to limited provision of dialysis and ESKD care in remote locations, some Aboriginal and Torres Strait Islander individuals with kidney disease are forced to relocate to a city to receive dialysis [34,37]; this dislocation has a profound social and emotional impact on patients and their families, communities, and spiritual connection [37] to Country. Leaving Country also manifests impacts on subsequent care pathways, including dental care [37].

This project focuses on oral health services research, using a culturally secure mixed methodology approach and a multidisciplinary team. The study will use a decolonizing [38] and interpretive [39] theoretical framework, grounded by a critical realist epistemology [40] and guided by an advocacy perspective.

### Study Aims

The overall aim of this study is to improve access, provision, and delivery of culturally secure dental care for Aboriginal and Torres Strait Islander individuals with kidney disease in South Australia. In this project, cultural security is understood as a doctrine that moves beyond cultural awareness and cultural safety to directly link understanding and actions with policies and procedures that create processes automatically applied to all Aboriginal and Torres Strait Islander people from the first point of seeking dental care. Although distinct from cultural awareness and cultural safety, both are necessary foundations for attaining cultural security [41]. This study is based in South Australia and is designed to support Aboriginal and Torres Strait Islander individuals with kidney disease in improving oral health via culturally secure dental management strategies to a standard that will meet the eligibility criteria for kidney transplantation. By working in partnership with Aboriginal and Torres Strait Islander patients, ACCHS, Aboriginal health workers (AHWs), and other kidney disease and dental stakeholders in South Australia, this study will achieve the following objectives:

1. Investigate the facilitators of and barriers to providing oral health care to Aboriginal and Torres Strait Islander patients with kidney disease in South Australia, by capturing the perspectives of the following stakeholders: ACCHS, AHWs, dental service providers, and renal service providers
2. Investigate the facilitators of and barriers to maintaining oral health among Aboriginal and Torres Strait Islander patients with kidney disease
3. Facilitate access to and completion of culturally secure dental care for Aboriginal and Torres Strait Islander individuals with kidney disease and their families
4. Provide oral health promotion training for AHWs at each of the participating ACCHS, with a specific emphasis on oral health needs of patients with ESKD
5. Generate co-designed strategies to better facilitate access to and provision of culturally secure dental services for Aboriginal and Torres Strait Islander people living with dental disease

## Methods

### Study Design

#### Overview

**Figure 1:**
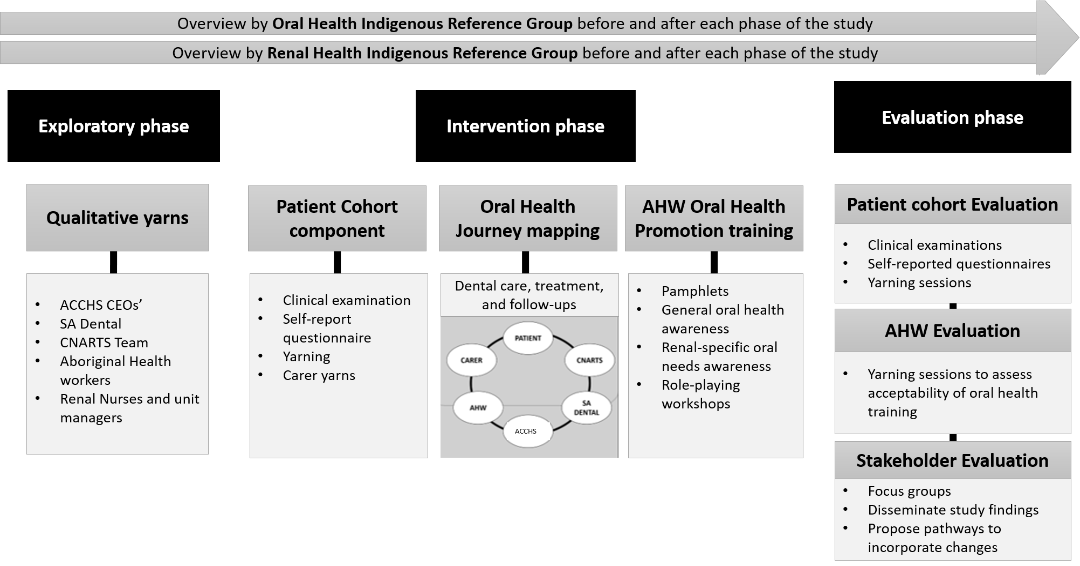
Project outline illustrating the 3 phases of the research project. ACCHS: Aboriginal Community Controlled Health Services; AHW: Aboriginal health worker; CEO: chief executive officer; CNARTS: Central and Northern Adelaide Renal and Transplantation Service; SA: South Australia.

This study is a result of community consultation and identification of the oral health care provision gap for Aboriginal and Torres Strait Islander patients with kidney disease in South Australia. This research collaboration is rooted in long-standing partnerships between the University of Adelaide’s Indigenous Oral Health Unit, 3 ACCHS (Yadu Health Aboriginal Corporation in Ceduna, Nunyara Aboriginal Health Service in Whyalla, and Umoona Tjutagku Health Service in Coober Pedy), South Australian Dental Service, Aboriginal Kidney Care Together – Improving Outcomes Now (AKction) group, and CNARTS. This study will be conducted over three consecutive phases: (1) exploratory phase, (2) intervention phase, and (3) evaluation phase. A schematic outline of the study design is presented in Figure 1.

#### Exploratory Phase

The exploratory phase will focus on further developing relationships with dental and renal stakeholders involved in the project and understanding existing oral health care pathways for Aboriginal and Torres Strait Islander patients with kidney disease. This phase will use a qualitative study design involving stakeholder interviews, focus groups, and yarning sessions (where culturally appropriate) [42-45]. The data gathered during the exploratory phase will inform multidisciplinary collaborative workshops between stakeholders, where stakeholders will be brought together to discuss and investigate potential organizational-level solutions to the burden of oral disease preventing Aboriginal and Torres Strait Islander patients from receiving kidney transplants in South Australia. In addition, this phase will provide rich and diverse narratives regarding the organizational experiences and challenges in accessing and facilitating oral health care provision and renal replacement therapy. This phase is a critical step toward building the foundation for the intervention phase of this project and developing a deep understanding of the practical and existing pathways of oral health care.

#### Intervention Phase

The intervention phase will use a mixed methods approach and will comprise 3 components: patient cohort, patient oral health journey mapping, and AHW oral health promotion training.

The patient cohort component will involve epidemiological clinical examinations and provision of culturally secure oral health care. Overall, three types of data will be collected during initial visits with participants: (1) clinical examinations to assess periodontal disease, according to American Academy of Periodontology–2017–modified classification [46]; (2) self-report questionnaires to collect participant demographics, self-reported oral and kidney health indicators, and associated behaviors; and (3) participant yarns to generate an appreciation for past oral health experiences, related challenges, and current needs. Family members and carers will be invited to participate in the yarns, and there will be an optional self-report questionnaire for carers to complete. This initial visit with participants will end with a discussion about the necessary steps for organizing and obtaining comprehensive oral health care, as deemed complete when participants are satisfied with their oral health. By participating in this project, participants will receive oral health care at no cost, they will have the option to be accompanied by members of the research team at each dental visit, and their family members with oral health needs will also be invited to access these services. Execution of this step is the chief priority of the project, with focus on highlighting the culturally secure dental care pathways among community members. To ensure continuity of care, members of the research team will be in contact with each participant on an ongoing basis, with frequency determined by participant-defined need. All dental staff in the public sector in South Australia receive cultural competency training and are evaluated according to the South Australian Health Aboriginal Competency framework [47].

The patient oral health journey mapping component will document patient journeys from the first point of dental contact to weekly or monthly follow-up according to patient-defined or clinician-defined needs. Follow-up will include research team visits, dental appointments, phone calls, and emails to the patient, families, dental managers, and AHWs, as necessary, by research team members. Each patient will have a unique management strategy and oral health plan, which will be coordinated by an appointed research team member (SS, BP, or JH). Patient journeys will be overseen by the Aboriginal and Torres Strait Islander Advisory Group, CNARTS, and special needs dental specialists. Documenting patient oral health journeys will be used to elucidate patient-centered challenges and enablers for accessing culturally secure dental care for Aboriginal and Torres Strait Islander people with kidney disease in South Australia. This approach is patient-centered and aims to identify barriers and solutions through a patient lens in the context of primary care services and wide health systems. The documentation of patient oral health journeys will be key to generating real-time solutions to challenges experienced by participants supported by this project during the evaluation phase. Interweaving multiple perspectives will enable an increased understanding of the complexities and gaps in dental care service provision and help to facilitate the most responsive and achievable improvements that can be readily translated.

There is strong evidence that supports the role of AHWs in improving health outcomes and health service provision for Aboriginal and Torres Strait Islander communities in Australia [48,49]. AHWs aid Aboriginal and Torres Strait Islander patients in navigating mainstream services and provide cultural brokerage between Indigenous and Western understandings of well-being [48,50-52]. Relationships of trust are imperative to holistic identification of patient needs, appointment attendance, and use of health services; AHWs successfully build upon familiar relationships in health care settings to the benefit of patients [53]. Although AHWs have been successful in providing oral health education to mothers [54] and applying fluoride varnish to children’s teeth in New South Wales [55], the use of AHWs for oral health promotion in South Australia has been sporadic and poorly defined [55]. Given the significant burden of oral disease experienced by Aboriginal and Torres Strait Islander people, provision of oral health education must align with community values that best meet the needs of Aboriginal and Torres Strait Islander patients. As such, the research team will work in partnership with AHWs at each of the ACCHS to develop, pilot, and evaluate basic oral health promotion training. Owing to the nature of this project, there will also be a component specific to CKD-related and ESKD-related oral health needs.

The expected outcome of the intervention phase is to develop evidence regarding the implementation of culturally secure dental care, where patients and families are supported by a multidisciplinary team. These findings will substantiate the arguments for policy translation that demands access to culturally secure primary dental services. Ultimately, the intervention phase aims to improve the oral health status and, subsequently, the eligibility for kidney transplantation among Aboriginal and Torres Strait Islander individuals with kidney disease in South Australia.

#### Evaluation Phase

The evaluation phase will use a mixed methods approach and will be similar in design to the intervention phase, with patient cohort evaluations, AHW evaluations, and stakeholder evaluations. The patient cohort component will involve epidemiological clinical examinations and evaluation of culturally secure oral health care provision. Overall, three types of data will be collected during follow-up visits after 6 months with participants: (1) clinical examinations to assess any changes in periodontal disease, according to the American Academy of Periodontology–2017 classification system [46]; (2) self-report questionnaires to collect any changes in self-reported oral and kidney health indicators and associated behaviors; and (3) participant yarns focused on participant evaluation of and reflection on the experience of dental care provided through this project. Family members and carers will again be invited to participate in the yarns, and there will be an optional self-report questionnaire for carers to complete. The clinical examinations and self-report questions will be compared with baseline measures collected during the intervention phase to assess the differences in clinical oral health and self-reported oral health. Evaluation of the usefulness of oral health promotion training and tools provided to AHWs at each of the 3 partnering ACCHS will be collected via yarns, with focus on the acceptability and relevance of the training and ways to improve the usefulness of these sessions.

Evaluation workshops or focus groups will be conducted with key stakeholders engaged throughout the duration of the project, including Aboriginal and Torres Strait Islander people with lived experience of kidney disease, ACCHS representatives, South Australian Dental Services, and CNARTS to (1) disseminate findings from the mapping exercises, (2) revisit strategies proposed during the workshop in the exploratory phase, and (3) develop realistic and specific pathways to incorporate strategies developed through this project. The evaluation workshops will be critical to ensure the translation of key elements of culturally secure dental care into existing primary health service records and monitoring structures to improve oral health experiences and kidney transplant eligibility among Aboriginal and Torres Strait Islander patients with kidney disease in South Australia.

### Participants and Recruitment

All Aboriginal and Torres Strait Islander people with kidney disease living in South Australia are eligible for inclusion in this study. However, our recruitment strategies will focus on Whyalla, Ceduna, Coober Pedy, and Adelaide. As of November 2021, CNARTS is providing nephrological care to 157 Aboriginal and Torres Strait Islander people with ESKD in South Australia, with primary health care needs delivered by each patient’s ACCHS. All efforts will be made to recruit all 157 patients. Over the past 10 years, the research team has developed strong relationships with the ACCHS stakeholders across South Australia, and each of the 3 ACCHS sites have been involved in project design, grant submission, and ethics obtainment for this project. Recruitment of participants will occur concurrently with stakeholder communications and ACCHS visits during the exploratory phase. Recruitment will use a purposive sampling strategy [56] to identify eligible patients willing to participate in this research project, using existing networks; patients will be recruited by word of mouth. Project information will also be posted in high-traffic areas at each of the 3 partnering ACCHS and renal sites. To be included in this study, participants must identify as Aboriginal or Torres Strait Islander, be aged ≥18 years, and have been clinically diagnosed with CKD. Patients eligible for inclusion in this study with low English literacy or comprehension will be provided with the option to have a translator from the Aboriginal Language Interpreting Service. Individuals who meet the inclusion criteria but are not enrolled during the original recruitment period will not be eligible to participate in the follow-up phases.

### Ethics Approval and Consent

Ethics approval for this study has been obtained from the Aboriginal Health Council of South Australia Human Research Ethics Committee (04-21-936) and University of Adelaide Human Research Ethics Committee. All study participants, including family members or carers who participate in the study, will be required to provide written informed consent.

### Aboriginal and Torres Strait Islander Advisory Group

This study is governed by an Aboriginal and Torres Strait Islander Advisory Group, which will oversee project orchestration, intervention delivery, project evaluation, and knowledge dissemination of the study findings. The Aboriginal and Torres Strait Islander Advisory Group will provide cultural guidance on all aspects of the study, including community engagement, staff recruitment and training, data collection and management, and dissemination of findings appropriate to communities and ACCHS. Secondary governance will be sought from the AKction reference group, a research team led by Aboriginal and Torres Strait Islander individuals with ESKD and carers of those with ESKD, whose lived experiences are invaluable. The involvement of the AKction reference group in an advisory capacity will be especially critical in terms of translation of findings to policy and health services, as the AKction team has already been successful in improving kidney service delivery for Aboriginal and Torres Strait Islander individuals with ESKD in South Australia.

### Data Collection

#### Questionnaires

Quantitative and descriptive data pertaining to kidney disease diagnosis, comorbidities, quality of life, health behaviors (including alcohol, tobacco use, and oral health), and indicators of social determinants of health will be obtained through self-report questionnaires completed in the intervention phase and followed up during the evaluation phase. Data collection will be overseen by a senior Aboriginal researcher (JH) and conducted by a team of Indigenous and non-Indigenous researchers with experience in working with the ACCHS and communities partnering in this project. All participants will be supported to complete a questionnaire containing items on oral health–related quality of life, social and emotional well-being, and dental behaviors.

#### Clinical Examinations

Hard and soft tissue status in the mouth will be assessed by recording caries experience, periodontal disease indicators, and gingivitis through standardized oral epidemiological examinations based on national oral health survey guidelines [57]. All epidemiological clinical examinations will be led by an experienced oral health specialist with extensive experience of working in partnership with Aboriginal and Torres Strait Islander communities. Patient convenience and comfort will be prioritized, and clinical examinations will be performed at participants’ homes, ACCHS, or another location preferred by the participant. Didactic, clinical, and cultural security training for examining teams will be conducted during the exploratory phase, before baseline collection in the intervention phase, with refresher sessions provided throughout the duration of the project. Daily reflexive debriefing sessions will be standard practice for all team members conducting field work. Examiners will be tested in the field to estimate interexaminer reliability. Intraclass correlation coefficients for caries, gingivitis, and periodontal disease scores will be used.

#### Oral Health Interventions

Aboriginal liaison project officers of the oral health promotion team from the South Australian Dental Services will aid the research team with the facilitation of dental appointments at public dental clinics, mobile dental vans, or private services, when necessary. The research team will be responsible for keeping detailed records of dental appointments and experiences, as informed by participants, which will be collated into the oral health journey mapping component. The oral health intervention will be based on the technique described by Tonetti et al [58]. This involves removal of subgingival dental plaque biofilms by scaling, root planing, and removal of teeth that cannot be saved, following administration of local anesthesia. It will additionally involve the removal of dental caries in the dental hard tissues, including replacement of insufficient restorations, and comprehensive prophylactic cleaning with fluoride varnish. The intervention will be performed by registered oral health professionals who will be overseen by registered periodontal and special needs dental specialists. The oral health intervention will occur for each participant at the facilities provided by South Australian Dental Services (including mobile dental vans). Dental care will commence immediately after the baseline visit, and the research team will provide transportation and liaise with participants, families, and dental services to ensure patient satisfaction and security. Where desired, research team members will accompany participants during dental appointments and support them in advocating for their dental needs.

#### Qualitative Data

Participants involved in the cohort component of the study and their family members will be invited to share their dental journeys via yarning sessions using Dadirri (deep listening approach) [42-45,59]. Yarning is a culturally secure research methodology that prioritizes a reciprocal 2-way approach to information sharing and negotiating. Yarning works to reduce power dynamics in research settings by eliminating the formality of researcher identity and demanding engaged interactions between individuals who each assume the position of learner and knower. The specifics of various approaches to yarning are as diverse as the Aboriginal and Torres Strait Islander communities across Australia, but fundamentally, yarning is built on relationships that require responsibility and accountability between people [42-44]. The mechanisms for information sharing in yarning sessions include storytelling and narratives, which enable connection between personal experiences regardless of place, culture, or time [60-62]. Yarning sessions with participants will explore each individual’s oral health journey regarding experiences of kidney disease, family, community, and Country.

### Statistical Analysis

#### Overview

The prevalence, extent, and severity of dental diseases will be calculated using the decayed, missing, and filled teeth index; loss of clinical attachment for periodontal disease; and bleeding on probing for gingival disease or gum disease. Data from the 2017 to 2018 National Survey of Adult Oral Health [63], which includes nationally representative data for both Aboriginal and Torres Strait Islander and non-Indigenous population-level estimates, will be used as benchmark oral health indicators. General analysis will comprise chi-square test and student 2-tailed *t* test within the study sample and nonoverlapping 95% CIs when comparing with population estimates.

#### Qualitative Analysis

Qualitative analysis will be grounded by a critical realist [40] approach and will use decolonizing [38] and interpretive [39] theoretical frameworks. Reflexive thematic analysis will be used to analyze both stakeholder interviews and participant yarning sessions, as guided by the framework by Braun and Clarke [64-66]. Reflexivity in thematic analysis not only embraces research subjectivity but also challenges researchers to continually analyze and explore the ways in which their lived experiences are influencing the analysis of qualitative data [65]. Aboriginal and Torres Strait Islander leadership and consultation throughout the analytic process will be critical to ensure that data are interpreted in a way that honors participant experiences and reflects participant meaning. Data will be inductively analyzed, without a structured codebook, to provide space for engaged and organic identification of themes [64,66]. Data will be coded line by line using NVivo software (version 12.6.1; QSR International). Initial coding will remain close to the data and maintain participant wording; once initial coding of all transcripts has been completed, data will be revisited, similar codes will be aggregated, and data will continually be reconceptualized for an iterative thematic development process [64-66].

#### Patient Mapping

The oral health journey of each participant will be mapped against the National Aboriginal and Torres Strait Islander Kidney Clinical Guidelines [67-69] health standards and frameworks for comparison against current clinical standards and best practices. Data from clinical examinations in the intervention phase will be used to categorize participants according to the level of dental care needed (ie, routine dental care vs emergency care). Details from dental appointments, aspects of yarning sessions, and self-reported measures from questionnaires will be collated to represent the journey of each study participant. Each journey will be written as a unique case study and comparatively analyzed against experiences of participants with both similar and differing needs. Findings from the dental journal mapping will be used to tabulate dental journey quality improvement strategies, which will be discussed with stakeholders in South Australia.

It is a well-established fact that Aboriginal and Torres Strait Islander communities face multiple barriers while accessing and maintaining good oral health. A strength of this project is that it addresses these barriers at the individual or family, community, institutional, and organizational levels (Table 1). The methodology used to address the barriers at each level and which phase of the study will focus on those particular barriers are presented in Table 1.

**Table 1:** Scientific framework of the project, with methodology mapped into the levels of a socioecological model.

Level addressed	Methods used	Phase of study
Individual	Yarning Participant satisfaction with oral health Increased eligibility for kidney transplant	Intervention
Community	Oral health promotion workshops for AHW^a^ Oral health promotion resources for ACCHS^b^ (posters and pamphlets) CKD^c^-specific oral health resources for ACCHS (posters and pamphlets)	Intervention
Institutional	Advocating for and facilitating the provision of culturally secure oral health, in partnership with dental teams Advocating for inclusion of oral health specialist in the multidisciplinary team for renal disease management Increasing awareness in dental services about CKD-specific oral health care needs in Aboriginal and Torres Strait Islander patients	Intervention and evaluation
Organizational	Advocating for policy changes that align with project outcomes	Evaluation
Organizational	Identifying key stakeholders and relationships in CKD within SA^d^ Organizational-level barriers to oral health provision	Exploratory

^a^AHW: Aboriginal health worker.

^b^ACCHS: Aboriginal Community Controlled Health Services.

^c^CKD: chronic kidney disease.

^d^SA: South Australia.

## Results

The funding for this project is obtained from a Department of Health (Government of Australia) grant, which was acquired in February 2021. The exploratory phase including stakeholder interviews was initiated in November 2021, and participant recruitment commenced in February 2022. The project has recruited 35 participants by October 2022, and it is estimated that a total of 40 to 42 participants will be recruited into the study according to the inclusion criteria specified in the grant. The first results (baseline) are expected to be submitted for publication in December 2022. The estimated date of project completion, along with the completion of 6-month follow-up with participants, is April 2023.

## Discussion

This study presents a unique opportunity to make significant gains in primary health care to not only improve oral health of Aboriginal and Torres Strait Islander communities of South Australia with kidney disease through timely and culturally secure dental care but also to reduce the burden of chronic disease that defines morbidity and mortality in this group. Improved oral health will lead to increased eligibility of Aboriginal and Torres Strait Islander people of South Australia with kidney disease to receive a kidney transplant, which improves longevity and quality of life for individuals, families, and communities. This study also aims to provide actionable strategies that can be translated into policy through stakeholder relationships and consultations. Evaluating the provision of culturally secure dental care will also contribute to cultural training for dental students. The evaluation of AHW oral health training will be used to enhance training resources developed for this project, which will be disseminated for use in all ACCHS across South Australia, through the Aboriginal Health Council of South Australia, thus creating more opportunities for dental education among communities.

Another strength is the use of interpretive and decolonizing theoretical frameworks and yarning as a methodology, all of which align with and respect Aboriginal and Torres Strait Islander values. An essential component of this project is the advocacy perspective, which aims to yield real-time results for participants and develop realistic strategies for policy implementation through stakeholder consultation. Finally, the foundational multidisciplinary relationships and engagement between the research team and the strong Aboriginal and Torres Strait Islander leadership will ensure that the project continually strives to meet community needs, thus improving the well-being of all those who are engaged in the study.

## Abbreviations

ACCHS: Aboriginal Community Controlled Health Services
AHW: Aboriginal health worker
AKction: Aboriginal Kidney Care Together – Improving Outcomes Now
CKD: chronic kidney disease
CNARTS: Central and Northern Adelaide Renal and Transplantation Service
ESKD: end-stage kidney disease

## Data Availability

The data sets generated and analyzed during this study are available from the corresponding author upon reasonable request. The authors confirm that supporting data and material in the study will be made available through Springer Nature’s Data Support Services, where possible.
